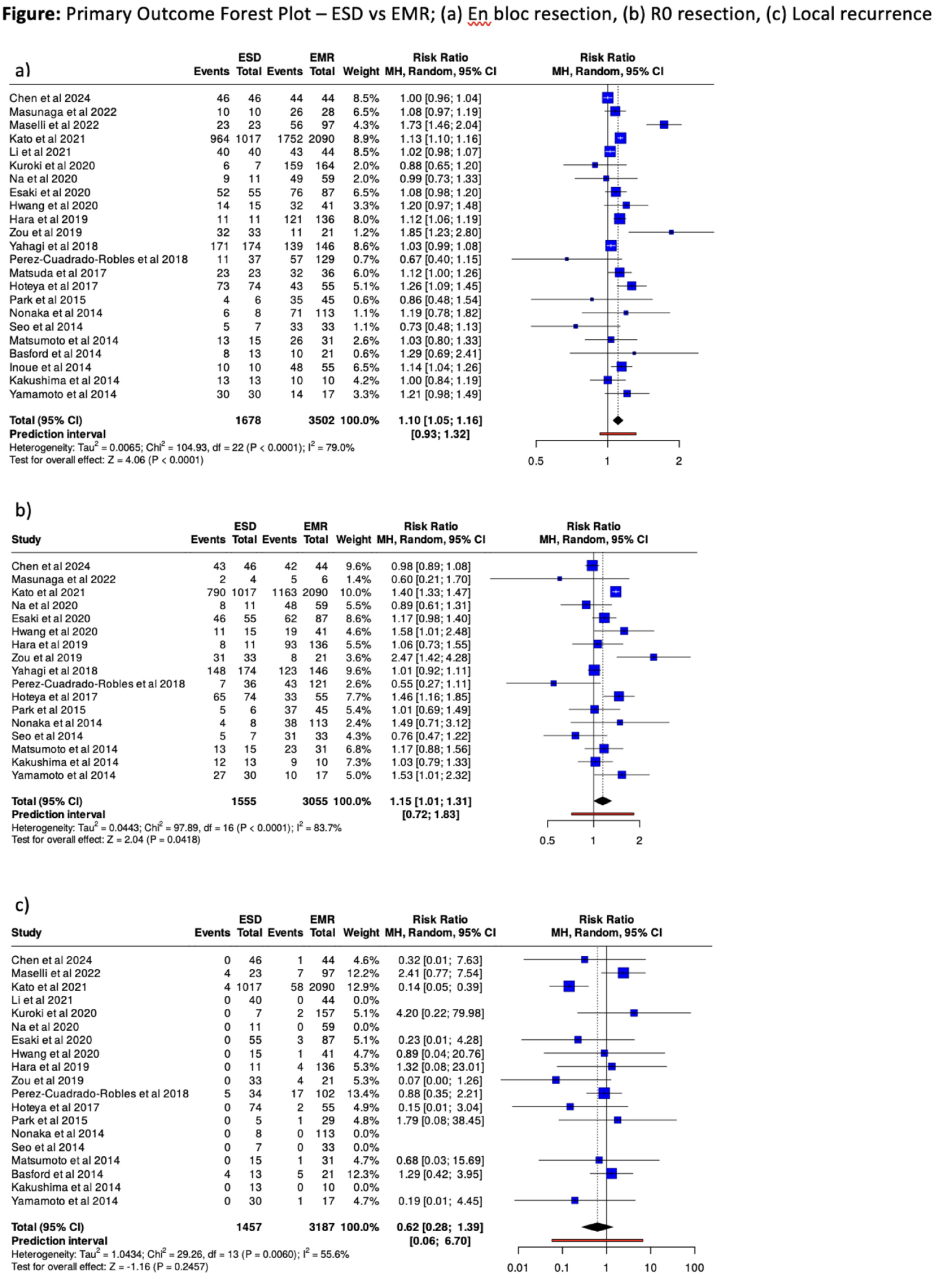# Poster Session I - A157 EFFICACY OF ENDOSCOPIC MUCOSAL RESECTION (EMR) VERSUS ENDOSCOPIC SUBMUCOSAL DISSECTION (ESD) FOR SUPERFICIAL DUODENAL LESIONS: A SYSTEMATIC REVIEW AND META-ANALYSIS

**DOI:** 10.1093/jcag/gwaf042.157

**Published:** 2026-02-13

**Authors:** H Li, T Nishimura, K Khalaf, Y Yuan, Y Fujiyoshi, M Hu, M A Bucheeri, C W Teshima, J Mosko, G May, N Calo

**Affiliations:** Medicine, Div of Gastroenterology, St Michael’s Hospital, Toronto, ON, Canada; Medicine, Div of Gastroenterology, St Michael’s Hospital, Toronto, ON, Canada; Medicine, Div of Gastroenterology, St Michael’s Hospital, Toronto, ON, Canada; McMaster University, Hamilton, ON, Canada; University of Ottawa, Ottawa, ON, Canada; Medicine, Div of Gastroenterology, St Michael’s Hospital, Toronto, ON, Canada; Medicine, Div of Gastroenterology, St Michael’s Hospital, Toronto, ON, Canada; Medicine, Div of Gastroenterology, St Michael’s Hospital, Toronto, ON, Canada; Medicine, Div of Gastroenterology, St Michael’s Hospital, Toronto, ON, Canada; Medicine, Div of Gastroenterology, St Michael’s Hospital, Toronto, ON, Canada; Medicine, Div of Gastroenterology, St Michael’s Hospital, Toronto, ON, Canada

## Abstract

**Background:**

ESD has been established as a minimally invasive resection method for superficial neoplasms of the esophagus, stomach, and colorectum. However, the optimal technique for superficial non-ampullary duodenal lesions (SDLs) is uncertain because potential gains in resection quality with ESD may trade off against safety.

**Aims:**

This systematic review and meta-analysis aimed to evaluate the efficacy and adverse event rates of EMR versus ESD in adult patients with SDLs.

**Methods:**

We searched MEDLINE and Embase (via Ovid) to July 14, 2025, for experimental and observational comparative studies. Two reviewers independently screened/extracted data assessed risk of bias and graded the level of certainty (GRADE). Random-effects models yielded risk ratios (RRs) and 95% CIs. A prespecified subgroup analysis examined studies in which the EMR group reported mean/median lesion diameter ≥20 mm.

**Results:**

Twenty-four retrospective cohort studies (5,515 lesions: EMR 3,709; ESD 1,806) were included. ESD improved en-bloc (RR 1.10, 95% CI 1.05–1.16) and R0 resection (RR 1.15, 95% CI 1.01–1.31); local recurrence did not differ (RR 0.62, 95% CI 0.28–1.39). Procedural risk was higher with ESD, including intraprocedural perforation (RR 9.34, 95% CI 6.03–14.45) and delayed perforation (RR 5.82, 95% CI 3.09–10.96); delayed bleeding and the need for surgery or endoscopic reintervention were also increased. In the ≥20 mm subgroup, differences diminished; only intraprocedural perforation remained higher with ESD (RR 3.76, 95% CI 1.04–13.58). Certainty of evidence (GRADE) was low for efficacy (en-bloc, R0) and local recurrence, high for perforations, delayed bleeding, and surgical intervention, moderate for endoscopic reintervention, and low for mortality.

**Conclusions:**

For SDLs, ESD achieves better resection quality but at greater procedural risk. EMR is a reasonable default first-line strategy for most benign polyps, reserving ESD for lesions where en bloc histology would alter management and where ESD can be completed in expert centers. Randomized trials, particularly lesion-stratified studies, are needed.

**Funding Agencies:**

None